# Obesity and overweight in Bangladeshi children and adolescents: a scoping review

**DOI:** 10.1186/1471-2458-14-70

**Published:** 2014-01-22

**Authors:** Sholeh Rahman, Md Tauhidul Islam, Dewan S Alam

**Affiliations:** 1 Centre for Control of Chronic Diseases (CCCD), icddr,b, 68 Shaheed Tajuddin Ahmed Sharani, Mohakhali Dhaka 1212, Bangladesh

**Keywords:** Overweight, Obesity, Children, Adolescents, Prevalence, Bangladesh

## Abstract

**Background:**

Obesity and overweight in children and adolescents is an emerging public health concern alongside under-nutrition in low and middle income countries. Our aim was to conduct a scoping review of literature to ascertain what is known about childhood and adolescent overweight and/or obesity in Bangladesh.

**Method:**

Using the scoping review based on York methodology, a comprehensive search of published academic articles, conference proceedings and grey literature was carried out through PubMed, BanglaJOL, Google and Google scholar limited to English-written papers. We summarized prevalence, risk factors and health outcomes of obesity/overweight in young children and adolescents aged between 0 to 19 years old in Bangladesh and highlighted use of different reference standards to measure childhood obesity.

**Results:**

In total 21 studies met the inclusion criteria. Nearly all of the reviewed articles used data from cross sectional studies, while only two used case–control design. Overall thirteen studies (62%) were primary research and eight (38%) included secondary data. Studies indicated an increasing trend in childhood obesity over time. Prevalence ranged from less than 1% to 17.9% based on different reference standards, with higher percentage amongst urban children across different age groups and sexes.

**Conclusion:**

This review demonstrated paucity of comprehensive literature on childhood obesity in Bangladesh, which might be explored through population-based prospective studies based on strong methodology and uniform reference standards. Sustainable and scalable preventative measures targeting high risk groups are required to avoid further rise.

## Background

Obesity in children and adolescents is rising alarmingly and approaching epidemic proportion in many economically developed countries, particularly in USA, Canada, Australia and several European countries [1]. Likewise in developing countries this issue is emerging as a public health crisis. According to a recent report, out of an estimated 43 million obese children worldwide in 2010, approximately 81% were from developing countries, half of which (18 million) were reported to be living in Asia despite of huge burden of under-nutrition. By 2020, it is estimated that the global prevalence of childhood obesity will reach approximately 60 million [2].

Factors contributing to the rising levels of childhood obesity in developing countries include socio-economic development, changes in lifestyle characterized by physical inactivity and unhealthy diet, living patterns, as well as rapid epidemiological and demographic transition [3,4].

WHO (2013) considers childhood obesity as “one of the most serious public health challenges of 21^st^ century” [5]. It accounts for a wide range of psychosocial and medical consequences [6]. Lower self-esteem, social isolation, poor academic achievement and peer problems are the most apparent immediate consequences in obese children [7]. Obesity in children and adolescents predisposes them to and is causally linked with cardio-metabolic disorders such as hypertension, dyslipidaemia and insulin resistance which are well-established illnesses [8]. As overweight and obesity is likely to follow through into adulthood, in the longer term, there is a greater risk of developing cardiovascular diseases [9,10]. Obesity at a young age seems to have substantial impact on reducing life expectancy [11]. Evidence also suggests a link between obesity in young girls with potential menstrual disorders, hypertension in pregnancy and sub-fertility [12].

In Bangladesh, the context of obesity and overweight has been underexplored, more so amongst younger age groups. Understanding the current situation and trends will provide useful insights into its risk factors and will assist health professionals and policy-makers in decision-making and developing future research agenda. This paper explored the availability of literature on childhood and adolescent obesity/overweight and its determinants in Bangladesh through a scoping review which, unlike a systematic review, offers a much broader perspective in the respective field which makes it more appropriate method to assess the present situation of childhood and adolescent obesity in Bangladesh [13].

## Methods

A scoping review was performed based on the York methodology outlined by Arksey and O’ Malley from the University of York, United Kingdom [13]. The ‘York framework’ suggested five stages that we have followed for this review:

Stage 1: Identifying the research question

Stage 2: Identifying the relevant studies

Stage 3: Study selection

Stage 4: Charting the data

Stage 5: Collating, summarizing and reporting the results

**Figure 1:**
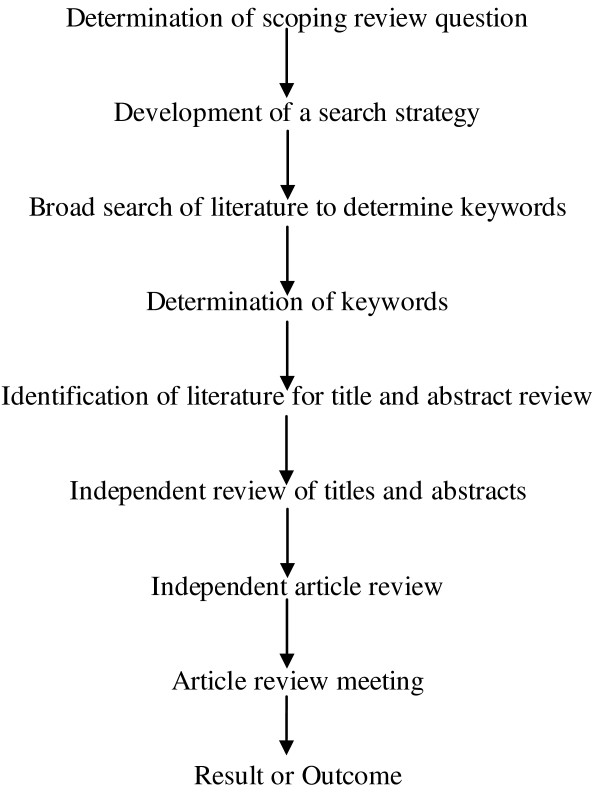
**Steps in performing the scoping review on childhood obesity in Bangladesh.** After determining the research question and search strategy, the authors conducted literature search using specific keywords followed by review of the selected literature to generate the results.

Initially, we defined research questions and developed search strategy and discussed them in a team meeting. Relevant literatures were then identified through a comprehensive search across different databases including Pubmed, Google, Google scholar and Bangladesh’s country specific search engine (BanglaJOL). We used specific key words that included obesity, overweight, adiposity, BMI, childhood, adolescents and Bangladesh. Search terms were combined using Boolean operators (AND, OR, NOT) to narrow the results [14]. In addition, hand searching of appropriate reference lists were undertaken to identify any additional literatures or grey publications. Figure 1 shows different steps of the review conducted.

### Selection of literature

We included the publication if:

• obesity and/or overweight was its primary or secondary outcome or an associated risk factor;

• study participants were Bangladeshi children and adolescents aged between 0 to 19 year old;

• it was relevant to any aspect of obesity, such as its prevalence, determinants, nutrition and growth in children and adolescents, anthropometry and health status; and

• it was written in English; no other limits on publication date of the articles were considered.

**Figure 2:**
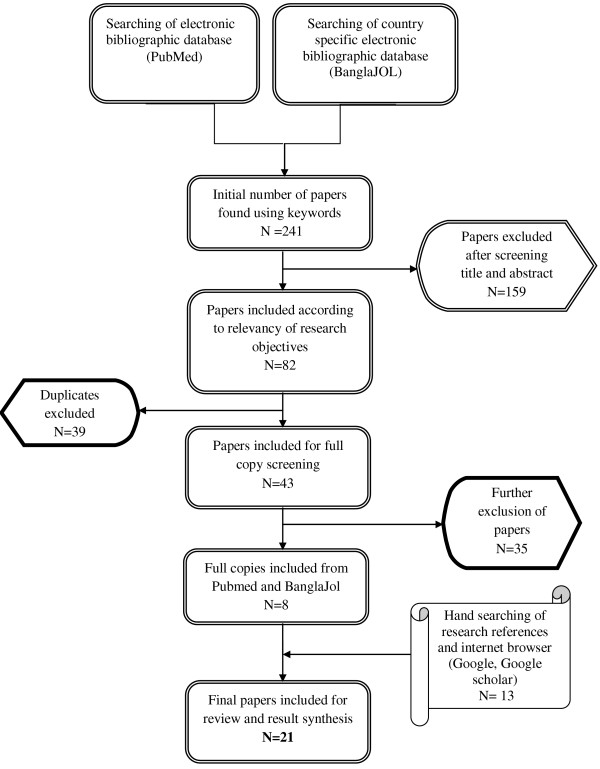
**Flowchart of the number of literature searched and selected.** Sequential steps undertaken for final inclusion of the literature in our review.

Papers were excluded if the study sample was older than 19 years of age or resided in countries other than Bangladesh. The initial search of databases revealed 241 literatures of which 159 were excluded after screening their titles and abstracts. One paper was not available even after contacting the library services and the publisher. The remaining 82 papers were screened for relevancy of their objectives, out of which a further 39 papers were excluded due to duplication or overlapping. Full copies of 43 literatures were read first independently and then reviewed again by the authors, which resulted in further exclusion of 35 papers as they did not meet our inclusion criteria. We retrieved 14 papers through manual search, from which one was excluded because of its apparently unrealistic results as well as lack of access to the full article [15]. The final number of papers included in this review narrowed down to 21, constituting of 19 full papers, 1 journal abstract and 1 conference proceeding [16-36] (Figure 2). Details of each of the included papers were then summarized (Table 1).

**Table 1:** Studies of childhood and adolescent obesity included in the review by study design, sample size, anthropometric indices and reference standards

**Author(s) and year**	**n**	**Study design**	**Anthropometric index**	**Reference standard**
Das et al., 2013 [16]	33,482	Cross-sectional	Weight for age (under 5) & BMI for age (5-19 years)	WHO, 2009^1^ National Obesity Observatory, 2011^2^
Bhuiyan, 2013 [17]	198	Case-Control	BMI	CDC, 2000^3^
Hossain et al., 2012 [18]	10,115*	Cross-sectional	BMI	WHO, 2006^4^
Mohsin et al., 2012 [19]	161	Cross-sectional	BMI	CDC, 2000^3^
Saha et al., 2011-2012 [20]	96	Cross-sectional	Not defined	N/A
Corsi et al., 2011 [21]	1439	Cross-sectional	BMI	WHO, 2004^5^
Jesmin et al., 2011 [22]	380	Cross-sectional	Weight for height	WHO, 2006^4^
Mohsin et al., 2011 [23]	161	Cross-sectional	BMI	CDC, 2000^3^
Mohsin et al., 2010 [24]	468	Cross-sectional	BMI	CDC, 2000^3^
Sultana, 2010 [25]	1200	Cross-sectional	Weight for height	WHO, 1995^6^
Kurshed et al., 2010 [26]	352	Cross-sectional	BMI	WHO, 1995^6^
Rahman et al., 2009 [27]	652	Cross-sectional	BMI	CDC, 2000^3^
Balarajan et al., 2009 [28]	1328	Cross-sectional	BMI	WHO, 1995^6^
Khan et al., 2009 [29]	425	Cross-sectional	BMI	WHO, 2004^5^
Sultan et al., 2008 [30]	172	Cross-sectional	BMI	Dietz & Robinson, 1998^7^
Shafique et al., 2007 [31]	16404	Cross-sectional	BMI	WHO, 2004^5^
BBS/UNICEF, 2007 [32]	3797	Cross-sectional	BMI	WHO, 1995^6^
Rahman et al., 2002 [33]	5000	Case control preceded by cross-sectional survey	Weight for Height	Not mentioned
de Onis et al., 2000 [34]	4787	Cross-sectional	Weight for Height	WHO, 1995^6^
Rahman et al., 1998 [35]	316	Cross-sectional	Weight for Height	WHO, 1993^8^
Ferdousi et.al., 2011 [36]	202	Cross-sectional	BMI	WHO, 2004^5^

^1^WHO Growth Standards for Infants and Young Children.

^2^A Simple Guide to Classifying Body Mass Index in Children, Public Health Observatories, England.

^3^2000 CDC Growth Charts for the United States: Methods and development.

^4^WHO Child Growth Standards.

^5^Appropriate body-mass index for Asian populations and its implications for policy and intervention strategies, report of a WHO expert committee.

^6^Physical status: the use and interpretation of anthropometry, report of a WHO expert committee.

^7^Use of body mass index as a measure of overweight in children and adolescents.

^8^Measuring changes in nutritional status.

^*^Overall sample size included women aged between 15 to 49 years, but sample size for the adolescent group was not mentioned.

## Results

Application of inclusion and exclusion criteria resulted in overall selection of 21 papers for this review; searching BanglaJOL, PubMed, Google and Google Scholar revealed 1, 7 and 13 relevant papers respectively. Literatures selected were from a 15 year period with the oldest from 1998 to the most recent in 2013. The search did not retrieve any relevant literature beyond these dates.

### Characteristics of the selected data

Seventy one percent of the studies (n = 15) included participants from both sexes and 29% involved females only (n = 6). Age range of the participants varied considerably across different studies. To make the interpretation easier, we decided to divide the studies into three categories according to their participants’ age range: group A (included those 5 years of age or younger), group B (included those over 5 years of age), group C (mixed group included subjects younger and older than 5 years of age). Accordingly twelve studies that examined obesity fell in group B, five in group A and only four in group C which included both age categories. In terms of the study site, majority of the studies (n = 14) were conducted in urban setting, six studies examined obesity in both urban and rural, and only one paper reported obesity in rural setting.

### Study design, measurement and reference standards

Sixty two percent (n = 13) of the studies were primary research and thirty eight percent (n = 8) used secondary data for their result synthesis. Bangladesh Demographic and Health Survey (BDHS 2004 and 2007), Nutrition Surveillance Project (NSP 2000–2004), WHO database (1996) and Diarrheal Disease Surveillance System (DDSS 1993–2011) were used as the sources of secondary data. The design of the selected studies was mostly cross-sectional, except for two studies which employed case–control design. Amongst the case control studies, one study was preceded by a cross-sectional survey. Different standards were used for defining overweight and obesity across the studies (Table 1). BMI cut-off value was used as a measure of overweight/obesity in 62% (n = 13) of the studies reviewed, including three studies in under 5 year age group. The remaining 28% (n = 6) adopted weight for height as an index of body size. Only one study did not mention the body size measurement index [20]. Another study used weight-for-age amongst children who were under 5 years of age and BMI-for-age amongst 5–19 years old [16]. No direct body composition based measure of adiposity was used in any of the reviewed articles. WHO standard for obesity and overweight has been changed and modified over time; however, it was the most commonly used reference for cut-off values in the studies. Few studies used CDC growth chart as their reference standard.

### Prevalence of obesity, associated factors and health outcome

Prevalence of overweight and/or obesity was reported in seventeen of the studies and it was the primary outcome in eight of them (47%). The rest of the studies addressed obesity as their secondary outcome. Since the studies did not use the same reference standards, the prevalence of overweight and obesity could not be compared across the studies. However, the reported prevalence varied widely and has been summarized according to the age groups and presented in Table 2. Recent survey by Bangladesh Bureau of Statistics (BBS) and UNICEF has reported 1.4% prevalence of overweight amongst children less than 5 years of age [32]. A study conducted amongst school children in Dhaka aged between 3 to 18 years of age, found 17.9% obese and 23.6% overweight children and adolescents [24]. In another study, the prevalence of obesity was found to be 13% and that of overweight was 11.5% amongst urban children aged between 2 to 10 years old; and obesity showed an increase with higher family income [35]. Most recent study (2013) among urban children, reported approximately a five fold increase in overweight and obesity over the past two decades [16]. In addition, two unpublished studies reported 17.8% and 7.6% obesity in children of different age categories [25,33]; obesity was significantly more in the highest income group (OR = 2.06) [25].

**Table 2:** Prevalence (%) of obesity and overweight amongst children and adolescents by sex, age range and setting of the study

	**Author(s) and year**	**Age range**	**Settings**	**Sex**	**Prevalence of overweight/obesity**
**Group A**	Jesmin et al., 2011	0-59 months	Urban	Both	Obesity: 3.9%
	Rahman et al., 2009	24-59 months	Rural	Both	At risk of overweight- male: 0.3%, female: 0.6%
					Overweight- male: 1.1%, female: 0.6%
	Sultan et al., 2008	1-12 months	Urban	Both	Obesity: 14%; Overweight: 25.6%
	BBS/UNICEF, 2007	0-59 months	Rural and urban	Both	Overweight/Obesity1.4% (male: 1.2%, female: 1.6%)
					Severe overweight/obesity: 0.3%
	de Onis et al., 2000	< 5 years	Rural and urban	Both	Overweight: 1.1%
**Group B**	Hossain et al., 2012	15-19 years^1^	Rural and urban	Females only	Obesity: less than 1%
	Corsi et al., 2011	15-19 years^1^	Rural and urban	Females only	Overweight: 1.6%
	Kurshed et al., 2010	10-18 years	Urban	Females only	Overweight/obesity: 4.6%
	Sultana, 2010	6-13 years	Urban	Both	Overweight: 13.2%; Obesity: 17.8%
	Khan et al., 2009	13-19 years^2^	Urban	Females only	Overweight: 2.1%; Obesity: 0.9%
	Balarajan et al., 2009	15-18 years^1^	Urban	Females only	Overweight/obesity: 1.8%
	Shafique et al., 2007	15-18 years^1^	Rural and urban	Females only	Zero prevalence of overweight or obesity
	Ferdousi et al., 2011	6-10 years	Urban	Both	Overweight: 18.32% and higher among males (10.9%)
**Group C**	Mohsin et al., 2010	3-18 years	Urban	Both	Overweight: 23.6%; Obesity: 17.9%
	Rahman et al., 2002	2-10 years	Urban	Both	Obesity: 7.6%
	Rahman et al., 1998	2-10 years	Urban	Both	Overweight: 11.5%; Obesity: 13%
	Das et al., 2013	< 5 years^3^	Urban	Both	Overweight & obesity in < 5: 5.15%
		5-19 years			Overweight & obesity in 5-19 years: 6.7%

Group A: ≤5years of age.

Group B: >5 years of age.

Group C: Mixed group including subjects younger and older than 5 years of age.

^1^Age range in overall sample was between 15 to 49 years.

^2^Age range of overall sample was between 13 to 49 years.

^3^Overall sample also included > 19 years adults.

Only two studies assessed metabolic syndrome in children and adolescents. One of them reported metabolic syndrome in more than 36% of obese children and adolescents with greater number amongst girls [23]. Another one reported 17% IGT, 2% Diabetes Mellitus and 26% high total cholesterol among obese children and adolescents [19]. Majority of the studies (n = 14) assessed determinants and associated risk factors of obesity. Overall higher socio-economic status, parents higher education (maternal), higher rank occupation, parental obesity, urban residence and physical inactivity were found to be positively associated with overweight and obesity. One study suggested that vitamin A supplements (OR = 1.665) and mother’s higher education (OR = 1.98) were positively associated with healthy, non-obese children [27].

## Discussion

Obesity has been studied extensively in many developed countries, but in Bangladesh studies and data related to obesity in children and adolescents are relatively scarce. To our knowledge, this study is the first and only scoping review that has been conducted on the topic in Bangladesh which has employed a comprehensive search of published and un-published literature.

In our review, it was found that the prevalence of overweight and obesity varies significantly across the studies. However, lack of a uniform definition and reference standard makes it difficult to compare the data across studies and predict the trends accurately. We observed greater data scarcity from rural settings than urban, as majority of the studies represented urban children. Although, socio-economic stratification had not been defined in most of the studies, overall greater prevalence of obesity and overweight was reported amongst urban children and adolescents compared to those from rural backgrounds which clearly suggest socio-economic and lifestyle differences in two different settings. In one study overweight and obesity was stated as high as 23.6% and 17.9% respectively amongst affluent urban children [24]. On the other hand, less than 2% of rural children had been reported as being overweight [27]. These findings are in line with similar studies from neighboring India and Thailand which reported greater prevalence of obesity amongst urban children than rural ones [37,38].

Substantial amount of evidence is available on the occurrence of metabolic syndrome in obese children and adolescents. Several studies have reported an association between obesity with a wide range of complications in childhood as well as an increased likelihood of premature onset of chronic illnesses in later life [39-42]. Evidences show simultaneous rise in the prevalence of diabetes and overweight in children, which suggests their close inter-relationship [43-45]. However, little is known in this regard in developing countries, due to limited number of studies. In our review too, we came across very few studies which estimated the prevalence of metabolic syndrome in relation to childhood obesity [19,23], which made it difficult to predict an association between the two. More than half of the studies included in our review assessed the risk factors of childhood obesity/overweight, irrespective of their design. A positive association between obesity with higher socio-economic status, lack of physical activity and urban residence has been reported. These findings are in accordance with studies from other developing countries which identified the similar risk factors [46,47]. In terms of maternal education, findings were contradictory; one study reported a positive association between child obesity and higher maternal education [16], while another one reported no association [17]. Overall, in our country there is a knowledge gap regarding other risk factors of obesity such as gender, birth weight and socio-cultural factors.

Our review has some limitations. Unlike a systematic review, we did not assess the quality of the studies included in our review, therefore, there is a possibility that the prevalence of overweight and/or obesity might have been exaggerated or underestimated as a result of methodological constraints. The reference standards that have been used in the studies reviewed have not been consistent. Some information might be missing due to inaccessibility of certain databases. In addition, there might have been other unpublished studies that are not available online which could not be revealed. Moreover, information on certain groups, such as tribal or ethnic minorities, is missing. Nevertheless, the findings of this review provide useful insights for future research needs in this area.

## Conclusion

This review demonstrated that there is a noticeable published literature gap regarding child and adolescence obesity/overweight in Bangladesh. Although, in general, young age obesity is not considered as a major public health problem in Bangladesh yet, it is alarmingly high and on rise amongst certain groups, particularly amongst urban children from affluent households. Addressing the problem at its earliest could be achieved through identifying high risk groups and designing sustainable interventions which could be implemented on a larger scale to prevent further rise in overweight and obesity. In addition, determining the prevalence of metabolic syndrome amongst them might play a vital role in preventing socio-economic and public health burden in later life.

Implementing school-based programs that could be expanded to population level to promote physical activity and healthy food habits could have a potential impact. Furthermore, large scale prospective studies based on context-specific definition and reference standards of obesity in South-Asian population are required to enhance data comparability and prediction of future trends.

## Competing interests

The authors declare that they have no competing interests.

## Authors' contributions

SR, MTI and DSA contributed equally to reviewing the literature, interpreting the results, writing the final report and they are responsible for the final content of this paper. All authors read and approved the final manuscript.

## Pre-publication history

The pre-publication history for this paper can be accessed here:

http://www.biomedcentral.com/1471-2458/14/70/prepub
